# Is Routine Preoperative Esophagogastroduodenoscopy Prior to Bariatric Surgery Mandatory? Systematic Review and Meta-analysis of 10,685 Patients

**DOI:** 10.1007/s11695-020-04672-4

**Published:** 2020-05-28

**Authors:** Walid El Ansari, Ayman El-Menyar, Brijesh Sathian, Hassan Al-Thani, Mohammed Al-Kuwari, Abdulla Al-Ansari

**Affiliations:** 1 Department of Surgery, Hamad Medical Corporation, Doha, Qatar; 2 College of Medicine, Qatar University, Doha, Qatar; 3 Schools of Health and Education, University of Skovde, Skövde, Sweden; 4 Department of Surgery, Trauma and Vascular Surgery, Clinical Research, Hamad General Hospital, Doha, Qatar; 5 Clinical Medicine, Weill Cornell Medical School, Doha, Qatar; 6 Department of Surgery, Trauma and Vascular Surgery Section, Hamad Medical Corporation, Doha, Qatar; 7 Department of Bariatric Surgery, Hamad Medical Corporation, Doha, Qatar

**Keywords:** Preoperative, Esophagogastroduodenoscopy, Laparoscopic sleeve gastrectomy, Bariatric surgery

## Abstract

**Background:**

This systematic review and meta-analysis searched, retrieved and synthesized the evidence as to whether preoperative esophagogastroduodenoscopy (p-EGD) should be routine before bariatric surgery (BS).

**Methods:**

Databases searched for retrospective, prospective, and randomized (RCT) or quasi-RCT studies (01 January 2000–30 April 2019) of outcomes of routine p-EGD before BS. STROBE checklist assessed the quality of the studies. P-EGD findings were categorized: Group 0 (no abnormal findings); Group 1 (abnormal findings that do not necessitate changing the surgical approach or postponing surgery); Group 2 (abnormal findings that change the surgical approach or postpone surgery); and Group 3 (findings that signify absolute contraindications to surgery). We assessed data heterogeneity and publication bias. Random effect model was used.

**Results:**

Twenty-five eligible studies were included (10,685 patients). Studies were heterogeneous, and there was publication bias. Group 0 comprised 5424 patients (56%, 95% CI: 45–67%); Group 1, 2064 patients (26%, 95% CI: 23–50%); Group 2, 1351 patients (16%, 95% CI: 11–21%); and Group 3 included 31 patients (0.4%, 95% CI: 0–1%).

**Conclusion:**

For 82% of patients, routine p-EGD did not change surgical plan/ postpone surgery. For 16% of patients, p-EGD findings necessitated changing the surgical approach/ postponing surgery, but the proportion of postponements due to medical treatment of H Pylori as opposed to “necessary” substantial change in surgical approach is unclear. For 0.4% patients, p-EGD findings signified absolute contraindication to surgery. These findings invite a revisit to whether p-EGD should be routine before BS, and whether it is judicious to expose many obese patients to an invasive procedure that has potential risk and insufficient evidence of effectiveness. Further justification is required.

## Introduction

There is a debate about the utility of routine preoperative esophagogastroduodenoscopy (p-EGD) screening of patients undergoing bariatric surgery (BS) [1, 2]. The European and Italian national recommendations advocate the use of presurgery upper gastrointestinal endoscopy together with multiple biopsies in the work-up of patients; conversely, the American Society for Metabolic & Bariatric Surgery only recommends it in selected cases with symptomatic gastric disease [3–5]. Generally, the question of routine p-EGD has many clinical implications and significant financial repercussions [1].

Some evidence supports routine p-EGD among patients undergoing BS. The reasons include the weak correlation between the patients’ symptoms and p-EGD findings, that p-EGD is convenient, safe, applied easily [6–8], and p-EGD findings may alter the management and hence eliminate the future development of gastric pathology [9], or detect asymptomatic benign or pre/malignant lesions. Missing asymptomatic lesions in some BS where the distal stomach and/or duodenum is rendered unreachable by esophagogastroduodenoscopy could lead to missing some lesions in the bypassed stomach that p-EGD could have discovered [10–16]. Some authors endorse that all BS patients have p-EGD, as after surgery, the endoscope may not reach the gastric/duodenal mucosa [17]. In agreement, others recommended that all BS patients should have upper gastrointestinal endoscopy [8]. For some procedures (e.g., laparoscopic adjustable gastric banding and vertical banded gastroplasty), p-EGD could provide information that might influence the operative procedure, particularly due to upper gastrointestinal lesions that often require medical therapy [7, 18].

It remains contested whether routine p-EGD should be undertaken for all patients undergoing e.g., laparoscopic sleeve gastrectomy (LSG) [19]. Some authors support routine p-EGD in patients with upper gastrointestinal symptoms (symptomatic cases only) [3, 20, 21]. Others suggest a selective approach for asymptomatic cases, because of the weak clinical relevance of most lesions discovered on routine p-EGD, its cost, and invasiveness [22, 23]. Still, other research found that routine p-EGD in LSG might require further justification for asymptomatic patients due to its low utility in managing such patients in regions with low prevalence of upper gastrointestinal cancers [2]. Only 2% of asymptomatic patients had any abnormality detected at p-EGD, none of which affected their treatment plan, and hence a focus on symptomatic patients only can safely reduce p-EGD rate by 80% [24].

Others reported that most of the pathology identified at p-EGD among patients scheduled for gastric banding did not significantly influence their management; however, two early cancers were detected [25]. In addition, although obesity is a risk factor for gastroesophageal reflux and esophageal adenocarcinoma, research could not confirm a high prevalence of Barrett’s esophagus among 233 patients selected for laparoscopic gastric banding [26]. Likewise, the association between obesity and reflux remains controversial [27], and it is unclear whether BS impacts the advancement of gastro-esophageal reflux disease (GERD) [28]. Despite a somewhat inaccessible foregut after bypass surgery, the low gastric cancer incidence among Caucasians [29] may not demand routine p-EGD [30].

Opinions remain divided as to whether p-EGD should be undertaken for all BS patients. One position is that the “intuitive reasons to continue p-EGD screening of BS patients include endoscopic findings that optimize medical management for the healing of their BS in a substantial proportion of patients and/or the endoscopic findings in at least a few patients that alter or delay the surgery itself” (p. 712) [22]. Conversely, others recommended that standard p-EGD is not indicated, as many BS patients are screened in order to discover clinically significant abnormalities [11]. For example, in Turkey, none of the 755 LSG patients had macro/microscopic malignant pathological finding in the preoperative upper gastrointestinal endoscopy [31]. In Brazil, researchers did not perform routine p-EGD on 649 LSG patients and only did when patients complained of abdominal pain or dysphagia; however, even with these symptomatic complaints, most patients had no abnormal findings [32]. Across 93.2% of BS patients, p-EGD findings were negative or had no effect on the preoperative management or choice of surgery; thus, it might not be wise to expose morbidly obese patients to a routine invasive uncomfortable procedure that carries potential (although minimal) risk [21]. Hence, authors have raised the question: “We do not screen the general population for those minor esophagogastroduodenoscopy findings; so why should we do it on people planned for bariatric surgery?” (p. 414) [21]. Likewise, a comment on “Is esophagogastroduodenoscopy before Roux-en-Y gastric bypass or sleeve gastrectomy mandatory?” concluded that p-EGD had no value in prediction or prevention of postoperative complications [33].

Such inconsistency highlights a gap as to whether routine p-EGD is sufficiently justified for all BS patients, and inspired the current systematic review and meta-analysis of the significance of routine p-EGD screening in BS. To the best of our knowledge, there exists no systematic review of the English literature on the topic, and no meta-analysis has been undertaken to answer this important question. Globally, many upper gastrointestinal endoscopies are performed for inappropriate indications, and the overuse of healthcare negatively affects healthcare quality and places pressure on endoscopy services [34]. Therefore, the current systematic review and meta-analysis assessed the justifications as to whether p-EGD should be routinely undertaken for all BS patients.

## Methods

This systematic review and meta-analysis was conducted and reported according to the Preferred Reporting Items for Systematic Reviews and Meta-Analyses (PRISMA) Statement. The study was registered at the International prospective register of systematic reviews (PROSPERO CRD42020157596).

## Literature Searches

A systematic review was carried out using PubMed, Cochrane Central Register of Controlled Trials (CENTRAL), WHO International Clinical Trials Registry Platform, Cochrane Library, MEDLINE, Scopus, clinicaltrials.gov, and Google scholar electronic databases. We used the keywords “bariatric surgery” “Esophagogastroduodenoscopy,” “preoperative” [in Title/Abstract]. The medical subject headings (MeSH) terms used were bariatric surgery (All Fields) AND “Esophagogastroduodenoscopy” (MeSH Terms); bariatric surgery (All Fields) AND “preoperative AND Esophagogastroduodenoscopy” (MeSH Terms); bariatric surgery (All Fields) AND “preoperative OR Esophagogastroduodenoscopy” (MeSH Terms). Additional searches were conducted using the reference lists of studies and review articles for a selection of relevant articles. The references of all included articles or relevant reviews were cross-checked.

## Inclusion/Exclusion Criteria

The inclusion criteria were (1) original studies, (2) English language, (3) published from 01 January 2000 through 30th April 2019, (4) assessed “Esophagogastroduodenoscopy” and “bariatric surgery,” and, (5) patients of any age, gender, and ethnicity. Articles other than original studies such as commentaries, letters to the editor, reviews, case reports, and studies that did not include outcomes or comparisons were also excluded. The consensus on the inclusion/exclusion criteria was premised on the fact that whether a given study provided information on the association between p-EGD and post-operative outcomes among bariatric surgery patients. Therefore, even studies with smaller sample sizes were also included in the initial evaluation. Three authors independently abstracted the data.

## Objectives

To assess the significance of routine p-EGD screening in BS, the specific objectives were to:

- Conduct a systematic review of the literature in order to identify all relevant articles on the topic;

- Employ Sharaf et al.’s classification [6] of predetermined criteria to categorize the p-EGD findings of each article into the four groups (detailed below);

- Compute the yield of p-EGD findings of each article in terms of the four groups of Sharaf et al.’s classification [6]; and,

- Use the findings emerging from the meta-analysis to make informed judgments of the justification as to whether p-EGD should be routinely undertaken for all BS patients or otherwise.

## Categorization of P-EGD Findings

In order to gauge the value of routine p-EGD screening in BS, we employed Sharaf et al.’s classification [6] of predetermined criteria to categorize p-EGD findings into four groups:

- Group 0: no abnormal p-EGD findings, i.e., normal.
- Group 1: abnormal p-EGD findings that do not necessitate changing the surgical approach or postponing surgery (e.g., mild esophagitis, gastritis and/or duodenitis, esophageal web).
- Group 2: abnormal p-EGD findings that change the surgical approach or postpone surgery (e.g., mucosal/submucosal mass lesions, ulcers, severe erosive esophagitis, gastritis, and/or duodenitis, Barrett’s esophagus, Bezoar, hiatal hernia, peptic stricture, Zenker’s or esophageal diverticula, arteriovenous malformations).
- Group 3: p-EGD findings that signify absolute contraindications to surgery (e.g., upper gastrointestinal cancers and varices).

## Data Extraction

The titles of the research articles obtained from the initial database searches were screened and relevant papers were selected. Then the abstracts and full texts were reviewed according to the inclusion criteria for final selection. Three authors independently reviewed the studies based on the exclusion and inclusion criteria. Initially, titles of the studies identified from the search were assessed for inclusion. Titles approved by the authors were moved to abstract screening. If three authors rejected a study at this stage, it was excluded from the review. In the third stage, full text articles were screened for eligibility. Only those studies approved by the three authors were included in the review. Agreement between the authors on the quality of the articles ranged between 90 and 100%. All disagreements were resolved by consensus among the authors. Data extracted from the selected articles included authors, the origin of studies, source population, study settings and duration, inclusion/exclusion criteria, data sources and measurement, sample size, and the yield of p-EGD findings in terms of the four groups of Sharaf et al.’s classification [6].

## Methodological Quality

The methodological quality of the selected studies was assessed based on five STROBE criteria from the checklist, namely, study design, setting, participants, data sources/measurement, and study size. The STROBE checklist and the five criteria selected from the checklist were most relevant in the assessment of the methodological quality of observational studies in epidemiology (Table 1).

**Table 1:** Summary and quality assessment of eligible studies for the meta-analysis in the current review

Author ^*a*^	Procedure	Study design	Sample	Data collection	D ^*b*^	Country	Patients N	Female (%)	Age ^*c*^	Group 0	Group 1	Group 2	Group 3	H Pylori	H H^*d*^	S patients	STROBE
2001 Frigg [18]	LAGB	P	C	1996–2000	4	Switzerland	104	84	39	46	47	14	0	23	13	**—**	Complete
2002 Schirmer [40]	RYGB	R	C	1986–2001	15	USA	536	**—**	**—**	510	17	9	0	62/206	3	**—**	Complete
2004 Sharaf [6]	Multiple procedures	P	C	2000–2002	2	USA	195	**—**	**—**	20	19	93	0	**—**	78	**—**	Complete
2006 Azagury [41]	LRYGB	R	C	1997–2004	7	Switzerland	319	82.1	40.4	172	33	65	0	124/318	54	**—**	Complete
2006 Korenkov [24]	LAGB	P	C	1997–2004	7	Germany	145	72.4	39.8	130	5	10	0	17/145	8	**—**	Complete
2006 Zeni [42]	LRYGB	R	C	2004–2005	1	USA	159	81.8	41.1	53	80	68	1	1/53	56	9	Complete
2007 Teivelis [43]	LRYGB	R	C	**—**	**—**	Brazil	42	87.5	42	**—**	26	2	0	25/42	**—**	**—**	Complete
2008 Al Akwaa [44]	Multiple procedures	R	C	2004–2007	3	Saudi Arabia	65	65	42	15	51	9	1	**—**	8	**—**	Complete
2008 de Moura Almeida [45]	Multiple procedures	P	C	2004–2005	1	Brazil	162	69.8	36.7	37	157	18	0	36/96	14	**—**	Complete
2008 Loewen [22]	Multiple procedures	R	C	2004–2006	2	USA	447	87	40.6	316	96	62	0	9/61	40	**—**	Complete
2008 Mong [9]	LRYGB	R	C	2000–2005	5	USA	272	87.1	43	**—**	37	10	1	**—**	**—**	40	Complete
2009 Munoz [14]	LRYGB	P	C	1999–2006	7	Chile	626	72.2	38.5	338	281	108	1	280/533	67	**—**	Complete
2010 Bueter [35]	LAGB	P	C	1997–2006	9	UK	68	85.3	34	**—**	33	22	0	**—**	22	**—**	Complete
2010 Küper [8]	Multiple procedures	P	C	Jan-Dec 2008	11 m	Germany	69	62.3	43.4	**—**	33	45	3	6/69	19	11/55	Complete
2012 Dietz [36]	Multiple procedures	P	C	**—**	**—**	Brazil	126	82.5	42.1	53	75	4	0	67/126	**—**	**—**	Complete
2012 Humphreys [25]	LAGB	P	C	2003–2010	7	UK	371	72.2	45	164	148	129	2	14/207	90	**—**	Complete
2013 D’hondt [37]	LRYGB	R	C	2003–2010	7	Belgium	652	70.9	39.5	208	437	208	2	115/652	159	**—**	Complete
2013 Peromaa-Haavisto [23]	LRYGB	P	C	2006–2010	4	Finland	412	50.5	**—**	191	95	117	1	41/412	87	**—**	Complete
2014 Gómez [38]	Multiple procedures	R	C	2006–2013	7	USA	232	82.3	51	**—**	98	78	4	8/232	55	**—**	Complete
2014 Petereit [39]	LRYGB	P	C	2010–2013	3	Lithuania	180	71.1	42.7	74	110	37	0	108/180	37	**—**	Complete
2014 Schigt [11]	Multiple procedures	P	C	2007–2012	5	Netherlands	523	76.7	44.3	257	0	0	1	84/523	**—**	**—**	Complete
2014 Tolone [28]	Multiple procedures	P	C	**—**	**—**	Italy	124	41.9	36	**—**	18	23	0	**—**	23	**—**	Complete
2016 Abd Ellatif [21]	Multiple procedures	P	C	2001–2015	4	Kuwait, KSA, Egypt	3219	79	37	2414	410	409	0	407/3219	383	**—**	Complete
2017 Lee [30]	Multiple procedures	P	C	2002–2014	12	China	268	**—**	**—**	138	109	74	14	58/243	48	**—**	Complete
2017 Salama [2]	LSG	R	C	2011–2014	3	Qatar	1369	69.7	35.65	675	550	144	0	597/1369	96	**—**	Complete

^*a*^Due to space limitations, only the first author is cited; *D* Duration of study; ^*b*^years; ^*c*^mean age in years; *HH* Hiatus hernia; ^*d*^number of patients; *S* Symptomatic; *P* prospective; *R* retrospective; *C* Convenience; *LAGB* Laparoscopic adjustable gastric banding; *RYGB* Roux-en-Y Gastric Bypass; *LRYGB* laparoscopic Roux-en-Y gastric bypass; *LSG* Laparoscopic Sleeve Gastrectomy; *m* months; *KSA* Kingdom of Saudi Arabia; — not reported

## Data Analysis and Synthesis

Prevalences were calculated for categorical variables. The decision to employ either a fixed-effect or random effect model depended on the results of statistical tests for heterogeneity. Data heterogeneity was assessed using the Cochrane Q homogeneity test (significance set at *p* < 0.10). If the studies were statistically homogeneous, a fixed-effect model was selected. A random effect model was used when studies were statistically heterogeneous. The Higgin’s *I*^2^ test is the ratio of true heterogeneity to the total variation in observed effects. A rough guide to interpretation of *I*^2^ test is 0–25%: might not be important; 25–50%: may represent moderate heterogeneity; 50–75%: may represent substantial heterogeneity; and > 75%: considerable heterogeneity. Publication bias was visually estimated by assessing funnel plots. Pooled estimates were calculated using the R 3.5.1 software.

## Results

**Fig. 1:**
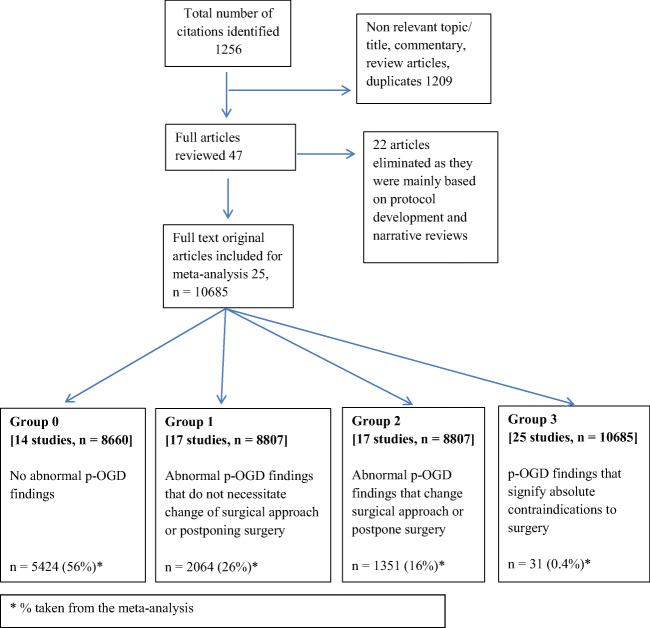
Flow diagram of study selection process for systematic review

The search generated a total of 1256 articles; 1209 articles were either non-relevant to the topic, duplicates, or review articles which were excluded. The relevant titles and/or abstracts and full text of the remaining 47 articles underwent detailed evaluation, after which 22 articles were further eliminated as these were mainly based on protocol development and narrative reviews. Finally, 25 original studies met all the review criteria and were considered for the final meta-analysis (Fig. 1 and Table 1) [2, 6, 8, 9, 11, 14, 18, 21–25, 28, 30, 35–45].

Median study duration was 4 years with an inter quartile range of 2–7 years. Overall average age was 40.7 years, and overall average percentage of males (25%) was lower than females (75%). All studies were non-randomized controlled trials, comprising 15 prospective and 10 retrospective studies. These studies had low or unclear risk of bias, unlikely to seriously alter the results. In addition, these studies had no serious risk of bias that can downgrade the quality. There was no inconsistency: the study populations were BS patients, and outcome assessment was consistent, namely the yield of p-EGD findings in terms of the four groups of Sharaf et al.’s classification [6].

## Outcome Measures

**Fig. 2:**
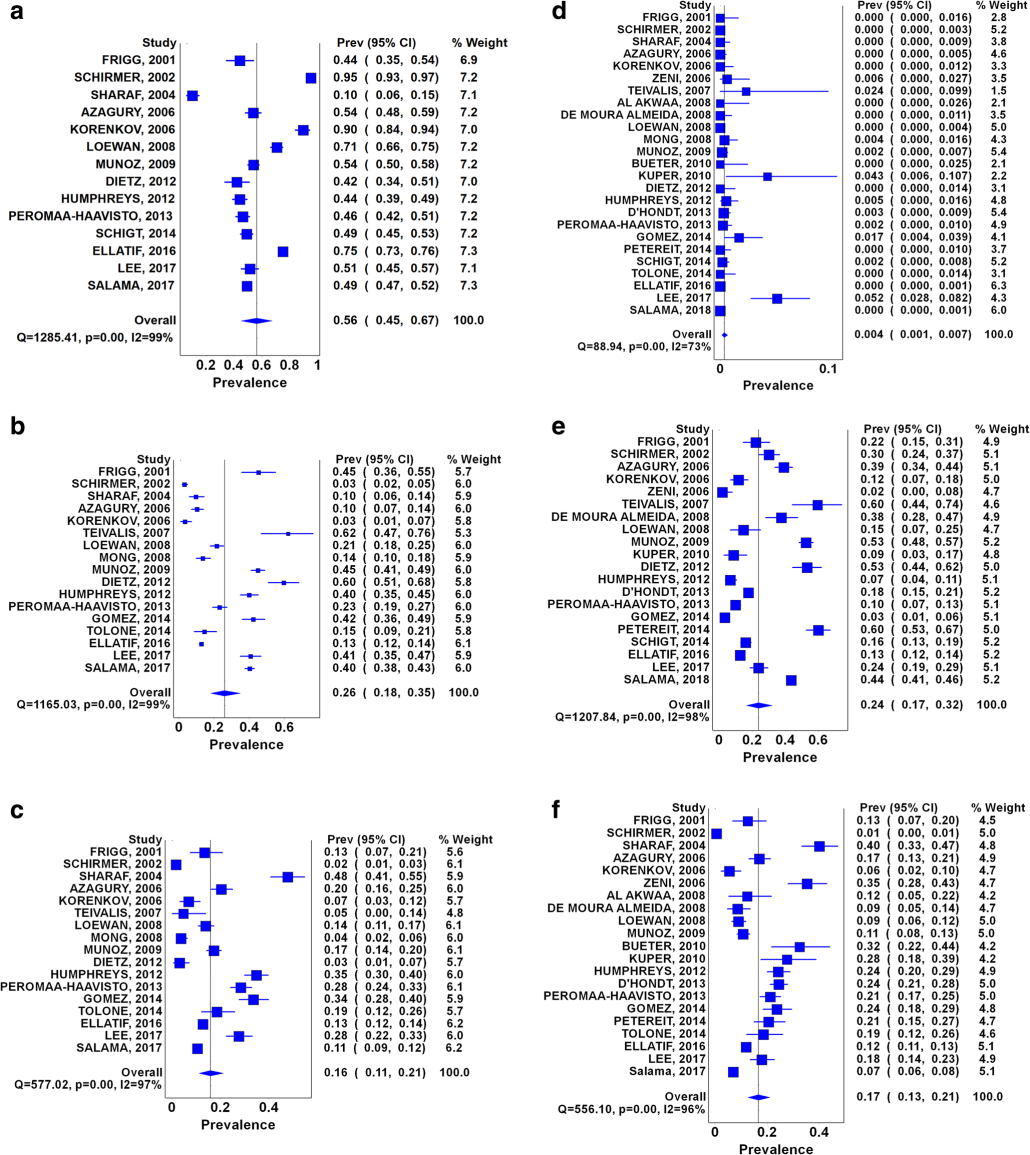
Forest plots of **a** no abnormal p-EGD findings (Group 0); **b** abnormal p-EGD findings that do not necessitate changing the surgical approach (Group 1); **c** abnormal p-EGD findings that change the surgical approach or postpone surgery (Group 2); **d** p-EGD findings that signify absolute contraindications to surgery (Group 3); **e** *H. pylori* infection; **f** Hiatal hernia

The total number of patients pooled was 10,685. Figure 2 depicts the meta-analysis of the 4 groups (groups 0–3) of patients based on their p-EGD findings. The largest group was Group 0 (no abnormal p-EGD findings, 56%, 95% CI: 45–67%) followed by Group 1 (abnormal p-EGD findings that do not necessitate changing the surgical approach or postponing surgery, 26%, 95% CI: 18–35%). These were followed by Group 2 (abnormal p-EGD findings that change the surgical approach or postpone surgery, 16%, 95% CI: 11–21%) and Group 3 (p-EGD findings that signify absolute contraindications to surgery, 0.4%, 95% CI: 0–1%). *H. pylori* infection was positive among about one-fourth of patients, and hiatal hernia was present in a mean of 17% of patients.

## Heterogeneity Among Included Studies

The results for the test of heterogeneity for the meta-analysis among bariatric surgery patients are displayed in the bottom line to the left of each Forest plot. For Group 0 (no abnormal p-EGD findings), *Q* [*χ*^2^] = 1285.41, *P* = 0.001, *I*^2^ = 99%, tau^2^ = 0.0159 (Fig. 2a); for Group 1 (abnormal p-EGD findings that do not necessitate changing the surgical approach or postponing surgery), *Q* [*χ*^2^] = 165.03, *P* = 0.001, *I*^2^ = 99%, tau^2^ = 0.140 (Fig. 2b); for Group 2 (abnormal p-EGD findings that change the surgical approach or postpone surgery), *Q* [*χ*^2^] = 557.02, *P* = 0.001, *I*^2^ = 97% tau^2^ = 0.077 (Fig. 2c); for Group 3 (p-EGD findings that signify absolute contraindications to surgery) *Q* [*χ*2] = 557.02, *P* = 0.001, *I*^2^ = 72%, tau^2^ = 0.007 (Fig. 2d); for *H* pylori infection *Q* [*χ*^2^] = 1207.84, *P* = 0.001, *I*^2^ = 98%, tau^2^ = 0.007 (Fig. 2e); and, for hiatal hernia *Q* [*χ*^2^] = 556.10, *P* = 0.001, *I*^2^ = 96%, tau^2^ = 0.196 (Fig. 2f). However, as *I*^2^ was > 25%, a random effect model was considered. Tau^2^ reflect the amount of true heterogeneity among the studies.

## Publication Bias and Funnel Plots

**Fig. 3:**
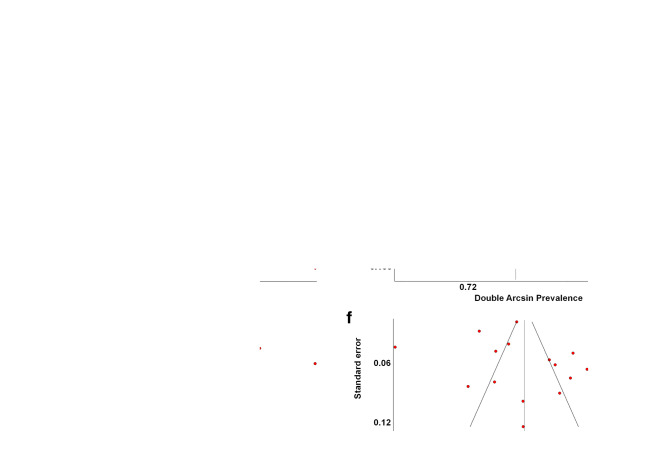
Funnel plots of **a** no abnormal p-EGD findings (Group 0); **b** abnormal p-EGD findings that do not necessitate changing the surgical approach (Group 1); **c** abnormal p-EGD findings that change the surgical approach or postpone surgery (Group 2); **d** p-EGD findings that signify absolute contraindications to surgery (Group 3); **e** *H. pylori* infection; **f** Hiatal hernia

For all of the above analyses, sensitivity analysis yielded consistent results. Based on a visual inspection of the funnel plots, there was evidence of publication bias for the included studies (Fig. 3). The funnel plots exhibited presence of studies with large standard error and they were not symmetrical.

## Limitation

The studies included in this meta-analysis did not report the frequency of multiple abdominal conditions. Rather, the studies reported the frequency of each abdominal condition separately. Hence, there might be a probability of multiple abdominal conditions for a single patient which would influence the overall estimation in Groups 1 and 2.

## Discussion

The current systematic review and meta-analysis is the first to assess the yield of p-EGD findings in terms of four groups [6], in order to gauge justifications as to whether p-EGD should be routine for all BS patients. Routine p-EGD can diagnose rare gastric pathologies [19]. The current review showed that 82% of patients had either no abnormal p-EGD findings (Group 0) or abnormal p-EGD findings that do not necessitate changing the surgical approach or postponing surgery (Group 1). Another 16% of patients required changing the surgical approach or postponing surgery based on the p-EGD findings (Group 2). Only 0.4% of patients had p-EGD findings that signified absolute contraindication to surgery (Group 3).

Generally, EGD carries risks to patients, as well as legal risks [46]. Hence, in addition to the p-EGD ‘yield’ in discovering/excluding pathologies, the appropriate gauging of whether routine p-EGD is justified for all BS patients needs to consider several parameters. These include the following: adverse effects of routine p-EGD; missing or over-diagnoses of lesions (false negatives, false positives); skill level of the esophagogastroduodenoscopy personnel; availability and cost of alternative (non-invasive) diagnostic methods to discover upper gastrointestinal pathology; and the costs of routine p-EGD. A related point is the changes that could occur to any missed pathology across time: i.e., initially before and then subsequent to BS (histological patterns of cellular alterations after gastric surgeries).

Adverse effects of esophagogastroduodenoscopy include infections, bleedings or perforations [47, 48], acute pancreatitis (direct trauma/gas insufflation) [49]; cardiopulmonary events [48]; methemoglobinemia (genetic predispositions/use of topical anesthetics) [50]; hypoxic respiratory failure/critical events requiring bronchoscopic intratracheal oxygen insufflation [8, 51]; orbital hematoma [52]; and Takotsubo cardiomyopathy with complete heart block [53]. Other effects include pre-endoscopy anxiety (unsedated esophagogastroduodenoscopy) [54], effects related to comorbidities of e.g., morbidly obese diabetic patients where the overnight fasting challenges the metabolic status, and sleep apnea (needs surveillance during sedation) [8]. Despite these, some authors suggest that the infrequent adverse events should not limit routine p-EGD [55].

As for missing important lesions (false negatives), the quality of the esophagogastroduodenoscopy varies [56]. In Spain, 17 out of 187 gastric cancer patients had prior esophagogastroduodenoscopy (9.1%), and 12 of those 17 missed gastric cancer had prior esophagogastroduodenoscopy with abnormal findings [57]. P-EGD is also frequently inaccurate at diagnosing hiatal hernia (particularly large hernias), where 23 patients undergoing sleeve gastrectomy had paraesophageal hernia intraoperatively; many of these patients were asymptomatic, and p-EGD revealed large hiatal hernia in only 4 patients [58–60]. Conversely, hiatal hernia repair was performed in 56 (5%) of patients positive for intraoperative findings despite a negative p-EGD for hiatal hernia [55]. A related point here pertains to the probability of changes of a given missed lesion, i.e., the changes of pathology across time and the histological cellular alterations after gastric surgeries [61]. Pre-surgery biopsies of 798 LSG patients showed non-significant findings in 86.2%; among them, 99.7% maintained a pattern without relevance for its follow-up; and some patients who had intestinal metaplasia reversed its histopathology (maybe following *H. pylori* treatment) [62]. Others found that the pre-operative inflammatory alterations were reduced post-operatively, where the chronic gastritis with inflammatory activity associated with *H. pylori* was reduced by 16.7%, and foveolar hyperplasia was reduced by 25% [61]. Further research can evaluate whether such improvements are due to treatment of *H. pylori* [61].

In terms of false positives, EGD over-diagnosed small hiatal hernias, most did not require repair, and 60% of EGD positive hiatal hernias were found to be negative intraoperatively [55]. Both the presence of symptoms and EGD findings may not always correlate with intraoperative findings [55]. In the current meta-analysis, p-EGD findings suggested hiatal hernia in a mean of 17% of patients (95% CI: 13–21%). However, the data provided by the studies does not enable one to speculate how many hiatal hernias/other lesions were missed or over-diagnosed during these EGDs.

In connection with the skill level, p-EGD has some subjectivity; hence, the endoscopist’s expertise could lead to over/under diagnoses [55, 63]. The endoscopist is vital in missed gastric cancer [57], and training/learning interventions could enhance the quality of endoscopy [63]. About 51.8% of the incomplete endoscopy reports did not have justification for its incompleteness [64]. Patients with no symptoms or no esophagogastroduodenoscopy evidence of hiatal hernias had hernia repairs (4%–6%), suggesting that small hiatal hernias are operator-dependent diagnoses [55]. The studies included in the current meta-analysis did not examine such skills, and we are unable to conclude how this might have affected the p-EGD yield we computed.

In terms of alternative diagnostic methods for gastric cancer pathologies, there are novel noninvasive screening techniques for e.g., Barrett’s esophagus [65] and *H. pylori* [66–68]. However, some authors might view that some novel techniques might be inferior to established gold standards, not all institutions might have advanced alternative diagnostic technologies, and esophagogastroduodenoscopy allows both the direct visualization and tissue biopsy [55].

Endoscopy is costly [1]. In the USA, the average hospital cost of an esophagogastroduodenoscopy with and without biopsy was $3732 and $3038 [69]. Endoscopy necessitates time, money, and personnel resources including experienced investigators, anesthesiological support, and special surveillance [8].

The current meta-analysis found that Group 2 patients (abnormal p-EGD findings that change the surgical approach or postpone surgery) amounted to 16%. However, it is not clear what proportion of these patients were postponed solely for *H. pylori* medical treatment as opposed to a “true” more substantial esophagogastroduodenoscopy-informed change in the surgical approach. This is important, as some might argue that if *H. pylori* is diagnosed by a non-invasive method (no need for esophagogastroduodenoscopy), and if the surgery waiting list time at a given institution is > 2–4 weeks (sufficient time for *H. pylori* treatment), then no postponement might have been required. One inquiry [2] examined the postponement, cancelation, or change of surgical approach based on the p-EGD findings across several sleeve gastrectomy studies and found that a considerable number of Group 2 patients were postponed solely for the treatment of *H. pylori*. This research [2] reported that across three studies, 21.5% [6], 12% [10], and 27% [30] of Group 2 patients had their BS postponed for *H. pylori* treatment, or waiting for *H. pylori* test result to assess severity of inflammation after medical treatment. Such findings suggest, that for the present meta-analysis, it might be reasonable to speculate that the proportion of Group 2 patients postponed due to a “true” change in surgical approach could be much less that the current 16%, further questioning the utility of routine p-EGD.

This review searched most of the citation databases and reference lists of the included studies. We also accessed paid articles. Nevertheless, a limitation of the current meta-analysis is that it included only published studies and only the English literature. We could not find “gray” literature, and hence, potential publication bias cannot be excluded. There were no studies from some regions of the world. However, 25 studies were included in this meta-analysis and we had a sizeable sample of 10,685 patients.

## Conclusions

The findings of this meta-analysis compel a revisit of current practice, and a re-evaluation of why p-EGD should be routine for all bariatric surgery patients. In 2016, about 634,897 bariatric operations were performed worldwide [70]. It might not be totally judicious to expose very large numbers of morbidly obese patients to a routine invasive uncomfortable procedure that has potential (although minimal) risk and insufficient evidence of effectiveness. Limitations include the lack of studies from some world regions and a small number of studies.
